# Introduction to Editorial Board Member: Professor David J. Mooney

**DOI:** 10.1002/btm2.10162

**Published:** 2020-05-11

**Authors:** Ovijit Chaudhuri

**Affiliations:** ^1^ Department of Mechanical Engineering Stanford University Stanford California USA

**FIGURE 1:**
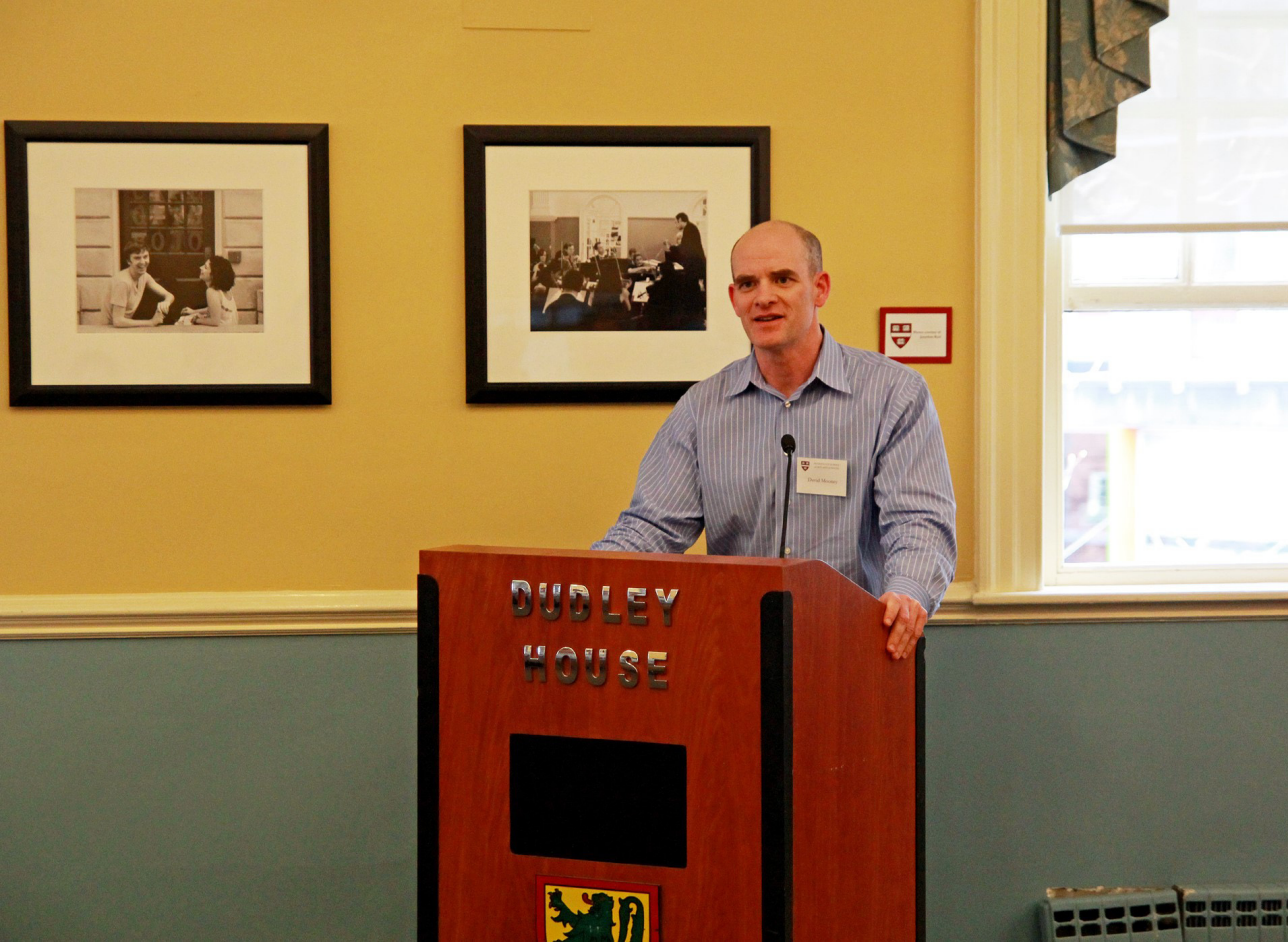
Professor Mooney after receiving the Everett Mendelsohn Excellence in Mentoring Award, given by the Graduate Student Council at Harvard University, in 2013. Photo courtesy of Dr Luo Gu

In this issue of *Bioengineering and Translational Medicine*, we are pleased to introduce our Editorial Board Member, Professor David J. Mooney (Figure 1). Professor Mooney is the Robert P. Pinkas Family Professor of Bioengineering at the John A. Paulson School of Engineering and Applied Sciences at Harvard University. He is a founding member of the Wyss Institute for Biologically Inspired Engineering at Harvard University, in which he serves as a core faculty member. He is a member of both the National Academy of Engineering and the National Academy of Medicine, and he is a fellow of the National Academy of Inventors. Professor Mooney is widely recognized for his influential work in biomaterials, drug delivery, tissue engineering and regenerative medicine, mechanotransduction, and immunotherapy. His publications have been cited over 90,000 times and include 13 papers with over 1,000 citations, his h‐index is 150, and he has given over 400 invited lectures. In 2019, *Nature Biotechnology* named him one of the top 10 translational researchers in biotechnology.

Professor Mooney earned his BS in Chemical Engineering at the University of Wisconsin, Madison. He then went on to conduct his PhD work in Chemical Engineering at the Massachusetts Institute of Technology, under the mentorship of Professor Robert Langer. After finishing his PhD, he worked as a postdoctoral fellow at Harvard University under the guidance of Dr Joseph Vacanti and Professor Donald Ingber. He started his career as a professor at the University of Michigan in 1994 and then moved to Harvard University in 2004.

In his early work, Professor Mooney made major advances in the use of biomaterials for regenerative medicine and tissue engineering. At the time, the paradigm in regenerative medicine had been the bolus delivery of single growth factors, which had limited efficacy. To address these limitations, Professor Mooney and others developed approaches to use biomaterial carriers for localized and sustained delivery of growth factors and other bioactive agents. His group demonstrated that modified, porous poly(lactide‐co‐glycolide) (PLG) scaffolds could deliver multiple growth factors with distinct kinetics to drive angiogenesis^1^ and bone formation, as well as deliver DNA‐encoding growth factors intracellularly to promote angiogenesis in vivo.^2^ They also developed a number of in vitro applications using these materials, including tissue‐engineered bone and models of tumors.^3^

Professor Mooney's early efforts also pioneered the use of alginate hydrogels for various biomedical applications.^4^ Alginate is a polysaccharide derived from algae, which forms a three‐dimensional (3D) nanoporous hydrogel when crosslinked with calcium that has similar structural characteristics to extracellular matrix. Alginate hydrogels are biocompatible, gel under mild conditions, and are injectable. Professor Mooney recognized and exploited these useful properties both in vivo and in vitro. His group showed how tuning various parameters, such as degradation and crosslinking, or applying mechanical perturbation can be used to control the spatiotemporal release of single or multiple bioactive molecules.^5^, ^6^ They utilized alginate gels to deliver a wide variety of bioactive molecules, including vascular endothelial growth factor and other heparin‐binding growth factors that naturally bind to alginate, as well as other bioactive molecules that must first be packaged or tethered to control their release. These approaches were used to promote angiogenesis, bone formation, and smooth muscle tissue formation in vivo. In parallel, they demonstrated that coupling the RGD (Arginine‐Glycine‐Aspartate) cell adhesion peptide sequence to the alginate allows cells to adhere to the otherwise inert gels.^7^ This enabled in vivo regenerative medicine applications involving infiltration of host cells into gels or delivery of exogenous cells, as well as two‐dimensional (2D) and 3D culture of adherent cells in vitro.

Professor Mooney's group continues to work on applying alginate toward therapeutic angiogenesis and regeneration of musculoskeletal tissues. Furthermore, they have continued to innovate with alginate, introducing various ways to modify the gels chemically and physically and expanding their use to new applications. Recent developments include alginate‐based tough gels^8^ and tough adhesives.^9^

Professor Mooney is also a leader in the field of mechanotransduction, the process by which cells sense and respond to mechanical cues. Professor Mooney's group has extensively characterized the mechanical properties of alginate gels and elucidated their underlying mechanisms; based on this knowledge, they have devised various approaches to modulate the mechanical properties of alginate‐based materials. In their early studies, they discovered that the stiffness of RGD‐coupled alginate hydrogels impacts cell proliferation, apoptosis, and differentiation in 2D culture, and they identified integrin clustering as a key mediator of mechanotransduction.^10^ They went on to show that hydrogel stiffness regulates the differentiation of mesenchymal stem cells in 3D culture^11^ and applied this finding to design a material that optimally promotes bone regeneration in vivo. Professor Mooney also recognized that tissues and extracellular matrices are typically not elastic but viscoelastic. His group developed alginate hydrogels with tunable viscoelasticity and showed that viscoelasticity, independent of stiffness, had a striking impact on various cell behaviors, including proliferation and stem cell differentiation, in both 2D culture and 3D culture.^12^ The role of matrix viscoelasticity in mechanotransduction has recently emerged as a major area of study in the field.

Professor Mooney is also a pioneer in the emerging field of immunoengineering, with a particular focus on cancer immunotherapy. In a seminal study, his group demonstrated that biomaterials could be used to develop potent cancer vaccines. PLG scaffolds delivering tumor‐specific antigens and danger signals were implanted in vivo to elicit a cytotoxic immune response against melanoma cells, representing the first therapeutic vaccine to eliminate melanoma tumors in mice.^13^ This technology recently completed a Phase I clinical trial in Stage IV melanoma patients. They have extended this approach to other types of cancer, as well as other biomaterial platforms, including alginate and a novel injectable mesoporous silica rod‐based system.^14^ The cancer vaccine technology is currently being commercialized by Novartis. Professor Mooney's group is also applying biomaterials to other areas in immunoengineering, such as promoting antigen‐specific tolerogenic responses, enhancing T‐cell regeneration after hematopoietic stem cell transplantation, and expanding T‐cells ex vivo.^15^

Beyond his scientific contributions, Professor Mooney has had a major impact at Harvard and in the broader bioengineering community through his service. He plays an active role in the National Academies and currently chairs Section 2 (Bioengineering) at the National Academy of Engineering. He serves as an editorial advisor to several journals and publishers, participates on several industry advisory boards, and serves on the visiting committees for a number of universities.

**FIGURE 2:**
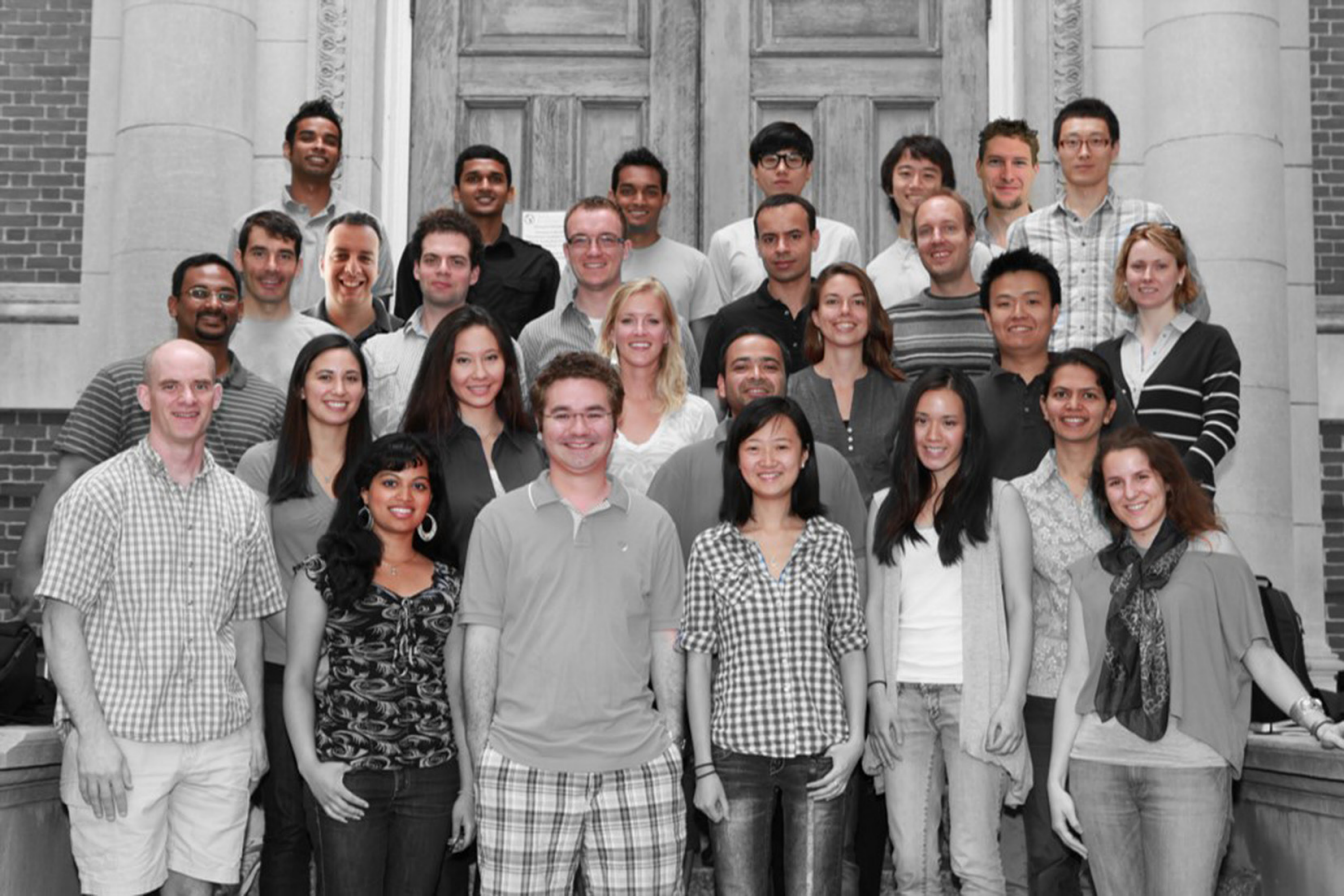
The Mooney lab group photo in 2012. Photo courtesy of the Mooney group

Last but not least, Professor Mooney is also a fantastic research mentor and role model to his trainees. He has trained 55 PhD students, 61 postdoctoral fellows, 25 M.S. students, and over 100 undergraduates in his laboratory (Figure 2). Despite having a large research group, he is deeply committed to mentoring each of his trainees and gives each of his trainees the support they need to pursue their individual research interests. Following his lead, members of the Mooney lab form a strong and supportive community, and many of us who go onto academic positions strive to emulate his mentorship style. Long after we leave his group, we continue to benefit from his advice and mentorship, as well as from the connection to the extensive network of Mooney alumni. Professor Mooney's excellent mentorship has been recognized by two major awards at Harvard: the Capers and Marion McDonald Award for Excellence in Mentoring and Advising at the School of Engineering and Applied Sciences and the Everett Mendelsohn Excellence in Mentoring Award bestowed by the Graduate Student Council. On behalf of his current and former trainees, I express my deep gratitude to Dave for the scientific opportunities we had with him and his mentorship and guidance.
